# *K*-Shortest-Path-Based Evacuation Routing with Police Resource Allocation in City Transportation Networks

**DOI:** 10.1371/journal.pone.0131962

**Published:** 2015-07-30

**Authors:** Yunyue He, Zhong Liu, Jianmai Shi, Yishan Wang, Jiaming Zhang, Jinyuan Liu

**Affiliations:** 1 Science and Technology on Information Systems Engineering Laboratory, National University of Defense Technology, Changsha, Hunan, China; 2 Beijing Special Engineering Design and Research Institute, Beijing, China; 3 Department of Management, National University of Defense Technology, Changsha, Hunan, China; Bangladesh University of Engineering and Technology, BANGLADESH

## Abstract

Emergency evacuation aims to transport people from dangerous places to safe shelters as quickly as possible. Police play an important role in the evacuation process, as they can handle traffic accidents immediately and help people move smoothly on roads. This paper investigates an evacuation routing problem that involves police resource allocation. We propose a novel *k*-th-shortest-path-based technique that uses explicit congestion control to optimize evacuation routing and police resource allocation. A nonlinear mixed-integer programming model is presented to formulate the problem. The model’s objective is to minimize the overall evacuation clearance time. Two algorithms are given to solve the problem. The first one linearizes the original model and solves the linearized problem with CPLEX. The second one is a heuristic algorithm that uses a police resource utilization efficiency index to directly solve the original model. This police resource utilization efficiency index significantly aids in the evaluation of road links from an evacuation throughput perspective. The proposed algorithms are tested with a number of examples based on real data from cities of different sizes. The computational results show that the police resource utilization efficiency index is very helpful in finding near-optimal solutions. Additionally, comparing the performance of the heuristic algorithm and the linearization method by using randomly generated examples indicates that the efficiency of the heuristic algorithm is superior.

## Introduction

Emergency evacuation has recently received extra attention due to an increase in both man-made and natural emergency events. The International Federation of Red Cross and Red Crescent Societies (IFRC) reports that 7,184 disasters occurred around the world during the first decade of the 21th century and that those disasters accounted for the deaths of more than 1 million people. These events affected approximately 2.55 billion people and incurred $986 billion in economic losses [1].The Sandia National Laboratory for the US Nuclear Regulatory Commission (NRC) states that an evacuation of greater than 1000 people happens approximately every 14–21 days [2]. Optimal emergency evacuation could reduce losses caused by these evacuation events.

Emergency evacuation is a process that displaces people from evacuation sources (i.e., dangerous places) and relocates them to evacuation destinations (e.g., safe shelters). Evacuation can happen on a scale as small as a building or as large as a city or country. Larger-scale evacuation, which creates routing problems in transportation systems, is our focus. One of the two purposes of this paper is to identify how to select an optimal egress solution for a given transportation network and assign vehicles to it. Compared to common location and routing operations, emergency evacuation presents some unique challenges:

- Evacuation tends to lack accurate supporting data. The emergency events often happen quickly and unexpectedly. Therefore, evacuation demand, information about transportation capacities and costs is incomplete (and may contain some noisy data) during the first stage of emergency events response [3].
- Evacuation demands overwhelm available evacuation transportation resources. For example, evacuation roads might be congested or blocked by numerous evacuees. It is hard to satisfy all evacuation demands, so trade-offs should be taken into consideration by the decision makers.
- Emergency evacuation operations require quick responses, which is important when designing an evacuation management system. In order to deal with emergent changes, the evacuation planning system needs to support expeditious mobilization.
- Evacuation implementation is also a difficult process; people in danger are often challenging to organize. Properly allocating organizational or management resources is vital in evacuation planning.

Police and patrols are supposed to play a significant role in evacuation. On one hand, the evacuation capacity of police is enhanced due to regular evacuation training. In the US state of Michigan, for example, both federal and state laws require government agencies to establish standard preparedness plans for emergency situations. These preparedness plans include emergency action team procedures, training on the emergency action plan, required evacuation drills, and details about which emergencies require evacuation [4]. Consequently, police are often the most reliable government resource, as they are expected to familiarize themselves with basic ideas of evacuation, especially regarding regulating traffic flow. If experienced operators organize an evacuation movement, the evacuation process should be smoother. However, the unified command and relatively comprehensive information that police can provide contribute to effective evacuation organization and coordination. Flustered people are less likely to follow coordinators unless they have established credibility. Therefore, police are the more suitable choice in this circumstance. Depending on how advanced their equipment is, highway patrol or state police may be capable of controlling highway access in order to move evacuees out of danger as soon as possible. Recognizing the aforementioned reasoning, an increasing number of governments and agencies address the role of police in evacuation guidance [5, 6]. Some researchers note that patrols or police could reduce the duration of an incident and thereby reduce congestion on the freeway [7, 8]. A natural solution would be to optimally deploy police across the road network for better evacuation efficiency. As a result, the second purpose of this paper is to optimally allocate police in order to improve the throughput of an involved transportation system.

We develop an integrated model for the evacuation routing and police resource deploying problem. The objective is to minimize the evacuation time of all evacuees to improve the efficiency of evacuation process. To approach this challenge, we employ the *k*-shortest-path method to describe the problem1[9–11]. The term “path” in this paper refers to the route from an evacuation source to an evacuation destination. Diverging from the transportation flow based model [12–15], this solution will assign an exact evacuation route to every evacuee. Doing so would reduce the difficulty of evacuation management when evacuees know their paths clearly. Additionally, optimally deploying police resources improves the transportation capacity of vital road links, which is significant in enhancing the efficiency of the entire evacuation.

The main contributions of this paper are as follows:

- A nonlinear mixed-integer programming (NMIP) model is developed to minimize overall evacuation clearance time, or the time which evacuees routing and police resource allocation decisions are considered. It mitigates the imbalance between numerous evacuation demands and limited evacuation resources.
- An index of resource utilization efficiency is proposed; it evaluates road links in the context of the whole evacuation. This index can be used for distinguishing the critical evacuation roads from the rest of a complex transportation network.
- A heuristic algorithm based on the police resource utilization efficiency index is presented to solve the NMIP model, which is an NP-Hard problem. The heuristic method can obtain near-optimal solutions to the problem in a reasonable computation time.
- A series of experiments are conducted to examine the effectiveness and efficiency of the heuristic approach. The performance of the heuristic is also compared with a linearization method assisted by CPLEX solver[16].

The remainder of the paper is organized as follows: in Section 2, relevant literature is reviewed; in Section 3, the formulation of the problem is presented; in Section 4, we describe two methods for solving the problem; Section 5 presents the computational experiments and discusses the results; and Section 6 concludes the discussion.

## Literature Review

In this section, we review the literature on emergency evacuation. This paper focuses on the disaster operation management area of the operation research and management science field. A comprehensive review of studies in this field can be found in the work of Altay and Green [17], although Galindo and Batta extended the review work [18].

The study of evacuation routing inside buildings began in the 1990s. [19, 20]. Chalmet et al. studied the large building evacuation problem by applying transshipment and dynamic network optimization models [21]. Their research also presented analysis of bottlenecks in building evacuation and had the potential to inform building design, building redesign, and building evacuability. Their analysis of bottlenecks inspired us to study the bottlenecks in a highway evacuation system.

The evacuation clearance time estimation, which is an important index for evaluating evacuation efficiency, has attracted much attention. Hobeika and Kim regarded the evacuee departure time distribution as an exponential distribution [22]. They employed a user equilibrium assignment algorithm to simulate traffic movements during evacuation. Highway structure and the number of vehicles involved were noted as significant factors in evacuation performance. To fill the gap between social research on population behavior and engineers’ contributions concerning evacuation models, Lindell and Prater explored the evacuation departure time distribution with an analysis of empirical data [23]. In his analysis of evacuees in a hurricane disaster, Lindell criticized the supposal of evacuees' behavior [24]. And Lindell described a simple, rapid method for calculating evacuation time estimates while accounting for evacuee behavior. Poisson process and Rayleigh distributions were also related to the evacuee departure time distributions [25, 26]. These remarkable studies have considered evacuation evaluation problems from diverse perspectives.

Evacuation in a transportation system is of particular interest. Yamada modeled the city as an undirected graph and assigned each resident to one of the places of refuge [15]. The author made use of the shortest path problem to obtain the shortest evacuation plan. Then, when capacity was introduced to the basic model, the minimal cost flow problem was adapted to modify the basic model. Cova and Johnson applied a network flow model to identify the optimal lane-based evacuation routing plans in a complex road network [12]. Their model tried to avoid traffic conflict at road intersections. Sherali and Carter thought that evacuation destination allocation was significant in the evacuation routing problem [27]. Shelter location problems were incorporated into their mixed-integer formulation, which aimed to minimize the total congestion-related evacuation time. In addition, an extraneous flow was superimposed on the network to represent traffic consisting of evacuees not using the designated shelters as destinations. Network-flow-based models, which are relatively macroscopic, are widely used in the transportation field. However, under the evacuation circumstances, each evacuee might want to be aware of his or her egress route from the beginning, rather than confirm the path at every intersection. This motivates us to build a model that specifies the evacuation route for each vehicle. Recognizing the evacuees’ needs in this way, Campos and Netto utilized the *k-*th shortest path to build a model aimed at large traffic capacity and shorter clearance time [28]. The *k-*th shortest path technique provides an evacuation plan with routes that remain intact from source to destination. Alexander and James utilized the M/G/c/c queuing theory for modeling while considering congestion and time delay [29]. The objectives of that model were reducing the clearance time, minimizing the total length of route and decreasing the ratio of traffic congestion. Their model based on *k-*th shortest path was presented as a way to generate an optimal route assignment for stochastic emergency evacuation planning. *MGCCSimul* simulation software was used in their study to measure the performance of evacuation policy. Inspired by their work, we suggest that congestion relief implemented by police is also a tremendously significant factor in improving the efficiency of evacuation. One of the objectives of our study is the optimal deployment of limited police on a transportation network to relieve the traffic pressure on certain roads. Now, we would like to take congestion into consideration when assessing the evacuation clearance time in a highway system. Some researchers have studied the evacuation with consideration of traffic congestion. Daganzo and So manage evacuation networks by using a non-anticipative, adaptive, decentralized strategy [30]. The authors think that people can and will use cross streets, and the cross streets will be used to divert traffic rom congested to underutilized routes, alleviating the effect. Kim et al. propose a contraflow reconfiguration method to minimize the evacuation time and reduce the congestion during evacuation [31]. They develop two heuristic method, namely, greedy heuristic and bottleneck relief heuristic to generate different solution according to different preference. As a supplement to these works, we would like to employee police to relieve traffic pressure.

To the best of our knowledge, existing studies rarely discusses police allocation issues, but there are some works concerning resource allocation in emergencies. Yan and Shih set up a time-space network model to minimize emergency maintenance time and rescue time [32]. Their model was a sophisticated multi-objective, mixed-integer, multiple-commodity network flow solution. A weighting method and a heuristic were adopted to effectively solve the problem. Considering the heavy damage to infrastructure in extreme disasters, Sarah et al. proposed an integrated optimization model based on network design and maintenance resource scheduling [14]. In this model, the maintenance resources could be scheduled to repair the destroyed roads to improve the traffic capacity. The object was to increase the cumulative flow during evacuation. The above studies gave us some insight on deploying highway patrol or police in order to improve the throughput, but there was still much work to be done to reach the goal.

The majority of emergency evacuation problems are complex and are usually NP-Hard problems [33]. With the development of nature-inspired methods and heuristic algorithms, many researchers took advantage of these new approaches to solve these complex problems [34]. To solve the large-scale continuous problem, Molina et al. came up with a local search chain algorithm called MA-SSW-Chains [35]. The Subgrouping Solis Wets’ algorithm was employed as their local search method. The algorithm does not explore all variables at the same time, however, it explores for a certain number of evacuations, a random set of variables. This random set of variables to change is maintained fixed during a certain number of evaluations. Then, a new random set is selected. Their study showed efficiency in solving high-dimensional continuous problems. Han and Zhang employed a genetic algorithm (GA) to obtain effective results for the emergency facility location and optimal allocation of emergency facilities problems [36]. Inspired by swarm intelligence approaches, such as the organization of bee colonies and task allocation among social insects, Santosa and Bazzan solved the distributed clustering and task allocation problem, and conducted a Robocup Rescue experiment [37]. The methods show superiority in solving sophisticated problems and the capabilty of coping with dynamically changing settings. Numerous researchers focused on the challenge of both modeling and solving algorithms in evacuation problems. In this paper, we develop two methods to solve our sophisticated problem by incorporating evacuation routing and police deployment.

There is an impressive body of literature in the emergency evacuation field. Researchers have studied assorted evacuation problems, and their achievements are inspiring. However, few papers discussed the evacuation routing problem with police resource allocation. Specifically, the police deployment problem (which is significant to organizing evacuation, maintaining order and improving throughput) was not the focus of anyone’s concerns. The effect of police on improving evacuation traffic is one of the important issues we managed to model. In this paper, we propose an optimal model that integrates evacuation routing and police resource allocation with road congestion considerations. The integrated optimization not only provides evacuees with clear egress paths but also enhances the traffic capacities of crucial roads by allocating police. To tackle the sophisticated NMIP problem, two algorithms are presented—a linearization method and a heuristic method.

## Model Formulation

In this section, the problem description, underlying assumptions and mathematic formulation are introduced.

The problem is a type of optimal routing problem (ORP) with consideration of resource allocation to improve relevant road capacities. We established a mathematical model that takes evacuation routing and police deployment into consideration. The objective of the model is to minimize the overall evacuation time. In our problem, a transportation network that consists of evacuation sources, destinations and related road links and intersections is given. The affected population is supposed to evacuate from sources to destinations through the network. Obviously, the traffic capacity of each road link is limited, and the capacity of road links is in measured by the vehicle arrival rate. Recognizing the effect of congestion and traffic incidents, our model assumes that the more vehicles are on a road, the more traffic incidents occur. When traffic accidents happen, the road is more likely to have a lower capacity because it leads to congestion. Police are considered a powerful force in ensuring smooth traffic on guarded roads, which ultimately contributes to the throughput of the transportation network. Due to varied conditions on each road, each road link requires a different number of police officers. The purpose here is to find a set of egress routes to evacuate the affected population over the transportation network and to allocate limited police to critical road links in order to improve transportation throughput. Our paper is concerned with the evacuation routing with police resource allocation (ERPRA) problem in city transportation networks. Some assumptions are proposed before introducing the mathematical formulation of this problem.

### Assumptions

The precondition of our research is that transportation network information is distinct. Furthermore, for clarity, we propose some rational underlying assumptions that highlight the nature of the problem. The hypothesis also states the boundaries of the problem we are concerned with.

1. The accident probability of each road link has a positive linear relation to the traffic flow on that road link.
  Many studies have focused on accident probability assessment [38–40]. Generally speaking, the more vehicles that a road holds, the more chances there are for an accident to happen (i.e., there is a positive relation between number of vehicles and accident probability). We would not like to focus on the accuracy of accident probability assessment. Therefore, to simplify assessment, a positive linear relation between vehicle numbers and accident probability is adopted in this paper, which would not lead to significant error.
2. There are two traffic states for each road, namely, accident state and non-accident state. A road guarded by police or patrols would be in non-accident state.

We presume that a road link has two different traffic capacities, one each for accident and non-accident circumstances. If there were no traffic incident, the capacity of a road would be larger. Otherwise, it would decrease considerably to a lower capacity. The actual capacity of a road link is an expectation value of two states and the accident probability. We make use of the road capacity expectation to depict the influence of traffic accidents and congestion. As mentioned, police are significant for improving evacuation efficiency. First, since they recognize the credibility of police, flustered people tend to perform better under police instructions. There seems to be much lower chance of traffic incidents around police. Furthermore, even if there were an accident on the road, police would address the incident as soon as possible. This helps reduce the congestion of road to some degree. Therefore, considering the positive effects of police, we suppose that patrols and police largely extend the traffic capacities of guarded roads.

### Notation

We propose nonlinear mixed-integer programming to describe the ERPRA problem. The notation used in the problem formulation is introduced below:

#### Indices

*i* an index for evacuation sources *S*_*i*_, *i* = 1,2,…,*I*, *I* is the total number of sources;

*j* an index for evacuation destinations *D*_*j*_, *j* = 1,2,…,*J*, *J* is the total number of Destinations;

*k* an index for *k*-th-shortest paths, *k* = 1,2,…,*K*, *K* is the total number of shortest paths for each pair of source and destinations;

*l* an index for road links *A*_*l*_, *l* = 1,2,…,*L*, *L* is the total number of road links;

#### Parameters

*N* the total number of nodes in the transportation network;

*e*_*i*_ the evacuation population from source *S*_*i*_;

*v*_*j*_ the population capacity of evacuation destination *D*_*j*_;

*t*_*ijk*_ the travel time through *k*-th shortest paths from source *S*_*i*_ to destination *D*_*j*_;

*α*_*lijk*_ 0–1 variable. 1 for the *k*-th shortest path from source *S*_*i*_ to destination *D*_*j*_ including road link *A*_*l*_; 0 for otherwise.

*λ*_*i*_ the evacuee arrival rate at source *S*_*i*_;

$\lambda_{l}^{L\text{max}}$ the maximum arrival rate expectation of road link *A*_*l*_;

*u*_*l*_ the maximum arrival rate of road link *A*_*l*_, when traffic accidents do not happen;

_*wl*_ the maximum arrival rate of road link *A*_*l*_, when traffic accidents have happened;

*p*_*l*_ current traffic accident probability of road link *A*_*l*_;

*r*_*l*_ the maximum traffic accident probability of road link *A*_*l*_;

*c*_*l*_ the police resource cost of road link *A*_*l*_;

*B* the total available police resources in the evacuation system;

#### Decision variables

*x*_*ijk*_ the proportion of evacuation population from source *S*_*i*_ to destination *D*_*j*_ through *k*-th shortest path;

*y*_*l*_ 0–1 variable. 1 for allocating police to road link *A*_*l*_; 0 for otherwise;

### Formulation

We formulated the problem as follows:

$$\left(\text{ERPRA}\right)\text{min}Z=\sum_{i}e_{i}\sum_{j}\sum_{k}x_{ijk}t_{ijk}$$ (1)

$$\text{S.T.}\sum_{j}\sum_{k}x_{ijk}=1,\;\forall i$$ (2)

$$\sum_{i}\sum_{k}e_{i}x_{ijk}\leq v_{j},\;\forall j$$ (3)

$$\sum_{i}\lambda_{i}\sum_{j}\sum_{k}\alpha_{lijk}x_{ijk}\leq \lambda_{l}^{L\text{max}},\;\forall l$$ (4)

$$\lambda_{l}^{L\text{max}}=y_{l}u_{l}+(1-y_{l})[(1-p_{l})u_{l}+p_{l}w_{l}],\;\forall l$$ (5)

$$p_{l}=\frac{\sum_{i}\lambda_{i}\sum_{j}\sum_{k}\alpha_{lijk}x_{ijk}}{u_{l}}r_{l},\;\forall l$$ (6)

$$\underset{l}{\sum }c_{l}y_{l}\leq B$$ (7)

$$0\leq x_{ijk}\leq 1,\;\forall i,j,k$$ (8)

$$y_{l}\in \{0,1\},\;\forall l$$ (9)

The objective function of the ERPRA formulation is the total evacuation travel time of all evacuees. Constraints (2) ensure that the entire population from source *S*_*i*_ is evacuated. Constraints (3) represent the aggregate capacity restrictions for destination *D*_*j*_. Constraints (4) ensure that the traffic flow on road link *A*_*l*_ will not exceed the road capacity. Constraints (5) show that the road capacity is an expectation value of traffic status, as noted in assumption 2). Constraints (6) reveal the positive linear correlation between traffic accident probability and the traffic flow on corresponding road link, as assumption 1) implies. Constraint (7) is the total police resource budget restriction. Constraints (8) specify the range of decision variables *x*_*ijk*_. Constraints (9) indicate that the decision variables *y*_*l*_ are 0–1 variables. Noting that the ERPRA formulation is NMIP, finding the optimal solution of the problem is complex.

## Solution Methods

In this section, we discuss two methods to solve the problem. The first one is a linearization method with a commercial solver, namely, CPLEX solver. The second one is a heuristic method based on the police resource utilization efficiency index of road links.

### Linearization method

The linearization method attempts to conform the non-linear ERPRA formulation into a linearized program by adding new variables and constraints. It ensures an equivalence property between the linearized formulation and the original one. CPLEX is good at solving the linear program (IP), which is used as a calculation tool in the solving process.

From the mathematics angle, the essence of $\lambda_{l}^{L\text{max}}$ is the function of *x*_*ijk*_, because $\lambda_{l}^{L\text{max}}$ partly depends on *p*_*l*_ (and $\lambda_{l}^{L\text{max}}$ is also partly influenced by *y*_*l*_), which is related to *x*_*ijk*_. In order to state the relationship more directly, we substitute constraints (5)(6) into constraints (4) and get the following:

$$(1+r_{l}-\frac{w_{l}}{u_{l}}r_{l})\sum_{i}\lambda_{i}\sum_{j}\sum_{k}\alpha_{lijk}x_{ijk}-(1-\frac{w_{l}}{u_{l}})r_{l}y_{l}\sum_{i}\lambda_{i}\sum_{j}\sum_{k}\alpha_{lijk}x_{ijk}\leq u_{l}$$ (10)

As Eq (10) shows, the ERPRA model is a NMIP. Because the quadratic coefficients matrix is not a positive semi-definite matrix, a commercial solver like CPLEX cannot handle it directly.

We employ the linearization technique introduced by Sherali and Alameddine, which replaces each nonlinear algebraic term [41]. The only nonlinear terms in the ERPRA model are *x*_*ijk*_ *y*_*l*_. We replace each *x*_*ijk*_ *y*_*l*_ with new variable *z*_*lijk*_, and new constraints are added to enforce *z*_*lijk*_ = *x*_*ijk*_ *y*_*l*_:

$$z_{lijk}\geq 0,\;\forall l,i,j,k$$ (11)

$$z_{lijk}\leq x_{ijk},\;\forall l,i,j,k$$ (12)

$$z_{lijk}\leq y_{l},\;\forall l,i,j,k$$ (13)

$$z_{lijk}\geq y_{l}+x_{ijk}-1,\;\forall l,i,j,k$$ (14)

The linearized evacuation routing with police resource allocation (LERPRA) formulation is stated below:

$$\left(\text{LERPRA}\right)\text{min}Z=\sum_{i}e_{i}\sum_{j}\sum_{k}x_{ijk}t_{ijk}$$

S.T.(2)-(3), (7)-(9), (11)-(14)

$$(1+r_{l}-\frac{w_{l}}{u_{l}}r_{l})\sum_{i}\lambda_{i}\sum_{j}\sum_{k}\alpha_{lijk}x_{ijk}-(1-\frac{w_{l}}{u_{l}})r_{l}\sum_{i}\lambda_{i}\sum_{j}\sum_{k}\alpha_{lijk}z_{lijk}\leq u_{l}$$ (15)

The LERPRA is a linearized mixed integer program, which can be solved by CPLEX. However, due to the added variables and constraints, solving the problem is time-consuming, which motivates us to search for a more effective and efficient method.

### Police resource utilization efficiency based heuristic method

The aim of this solving method is maximization of the efficiency of each police resource. In this paper, the term “bottleneck” means that the traffic flow on the road is close to the expected maximum arrival rate. We suppose bottleneck roads are critical to improving evacuation system throughput, because improving the overall throughput requires an increased flow on relevant roads, among which bottlenecks are the hardest to mitigate. Considering the definition of bottlenecks in this paper, potential room for improvement is limited without police presence at bottlenecks, and increased traffic flow would make passing these bottlenecks challenging. Rationally, in order to improve throughput, deploying police to some bottlenecks is an appropriate tactic. However, doing so does not guarantee that a throughput increase would be seen after allocating police to an arbitrary bottleneck. Careful bottleneck selection is needed to deploy police and improve network throughput. The main procedure of this heuristic method is assessing the effect of each bottleneck on throughput and deploying police officers to the most valuable one, which will help maximize the efficiency and effectiveness of police. We repeat the step iteratively until some terminal conditions are satisfied.

**Fig 1:**
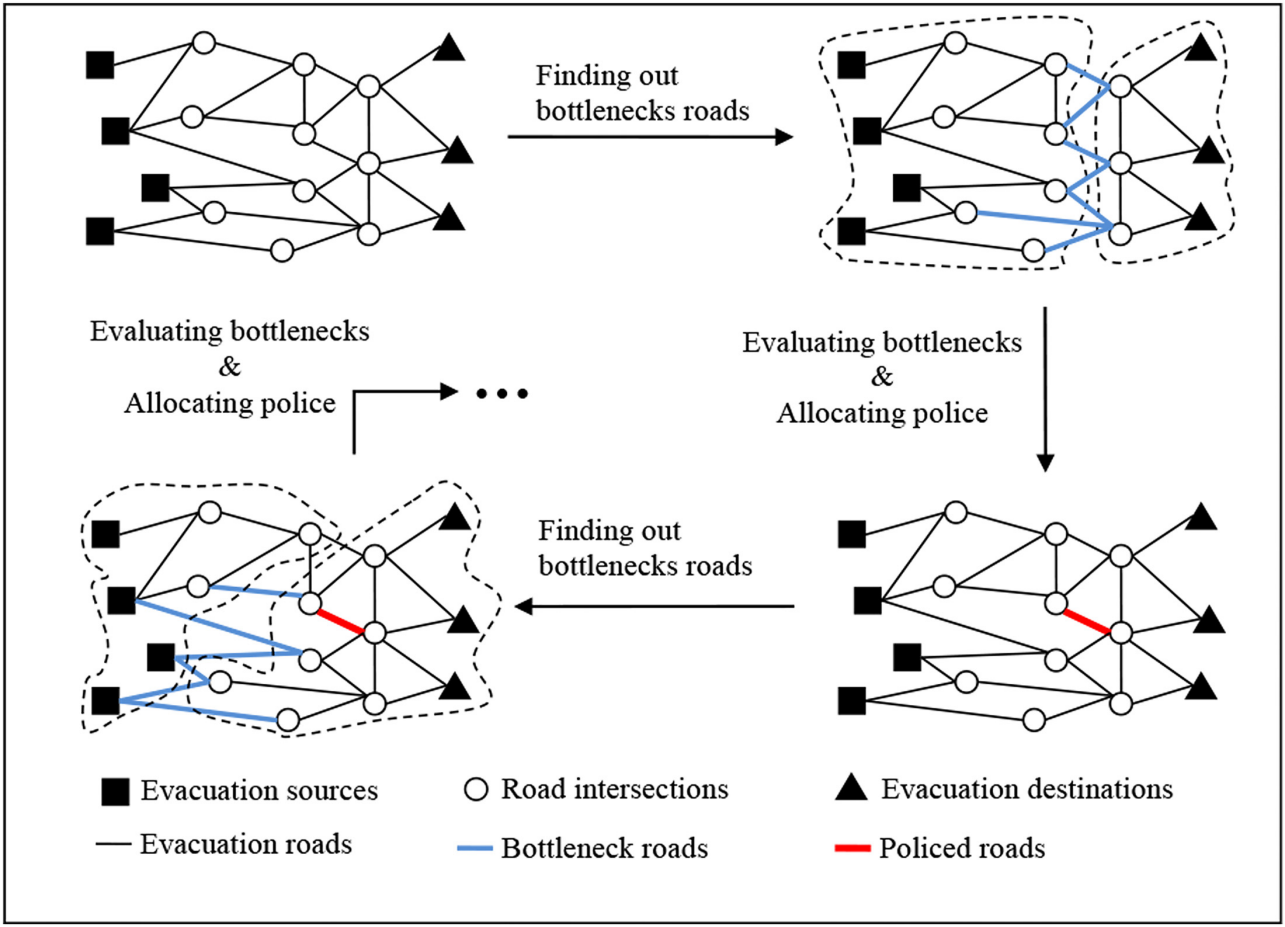
The motivation of the heuristic algorithm.

We describe the heuristic method in Fig 1. The main idea is to find the bottlenecked roads in each iteration, evaluate the bottlenecks, and allocate police resources to the most valuable roads. This should be executed in loops until either the evacuation situation does not improve or police resources are used up. The key step in the method is identifying the crucial road links rapidly and accurately.

The heuristic method based on police resource utilization efficiency contains two steps. In step 1, we solve a special case where police resource budget is zero. In step 2, leveraging the first step solution, police resources are allocated to the road links that are expected to be valuable.

#### Step 1: solving the no police resource ERPRA problem

If *B* = 0, then *y*_*l*_ = 0, ∀*l*, so constraints (7) and (9) can be eliminated, and constraints (5) should be modified as Eq (16):

$$\lambda_{l}^{L\text{max}}=(1-p_{l})u_{l}+p_{l}w_{l},\;\forall l$$ (16)

When no police resource is available, the ERPRA problem becomes a linear continuous program and can be solved by the Simplex Method [42].

#### Step 2: allocating police resources to valuable road links

This step improves upon the initial evacuation plan efficiency by allocating police to some important road links. Increasing traffic flow on the short paths is important in reducing the total evacuation time. Reductions in evacuation time are achieved by allocating police resources to the road links that are crucial to the shortest paths, so that the shortest evacuation paths can operate smoothly. A loop generates the improved evacuation plan. The loop consists of 4 procedures: (1) finding bottlenecked roads, (2) evaluating the bottlenecks, (3) allocating police to valuable roads, and (4) re-planning evacuation activity. Every time the loop runs, a better evacuation plan could be put in place and some police resources could be allocated. Step 2 will be finished when there is no better evacuation plan or no remaining police. After the adjustments from step 2, the evacuation plan could be much better than the no police resource plan.

The first procedure is finding bottleneck road links. In this procedure, we find bottlenecked road links in the current evacuation plan. As previously mentioned, the bottlenecked road links limit the increasing traffic flow of paths. If we allocate police resources to one of the bottlenecks, it might not increase the traffic flow on an evacuation path because there may be more than one bottleneck on any given evacuation path. The capacity of all bottlenecks on a path must be improved before traffic flow on the path will increase. Therefore, when allocating police to bottlenecks, we should pick out a set of bottleneck roads that are included in an evacuation path.

First, in order to determine if the candidate bottleneck road links are part of evacuation paths, we traverse all the road links to check which satisfy the constraints (17):

$$\lambda_{l}^{L\text{max}}-\sum_{i}\lambda_{i}\sum_{j}\sum_{k}\alpha_{lijk}x_{ijk}\leq \varepsilon ,\;\forall l$$ (17)

In constraints (17), parameter *ε* is the threshold value, which is used to ignore calculation error of computer. If a road link satisfies the constraints (17), the traffic pressure of that road link is high. These road links become the candidates for bottlenecked road links.

Second, we traverse each evacuation path and create a set where each path records the bottlenecks of that path. For each evacuation path satisfying *x*_*ijk*_ > 0, we check all road links on the path to determine if they are bottleneck candidates. The bottlenecked road links of a path are collected in the set. In the heuristic method, each path’s bottleneck set is a basic unit for allocating police resources.

The second procedure is evaluating bottlenecks. If we allocate police to the bottleneck set of an evacuation path, then the traffic flow on that path could increase. In this procedure, we need to evaluate all bottleneck sets. In other words, the main goal is evaluating the potential traffic capacity increase of evacuation paths. The police resource utilization efficiency index is designed to evaluate the road links from an overarching perspective. The evaluation index is based on the traffic flow increase and consideration of police resource costs.

We treat the potential traffic flow increase of an evacuation path as the performance of a bottleneck set. However, precisely calculating the potential flow increase after police allocation is too complex. Some estimations should be done for evaluating the potential increase to evacuation paths. Formula (17) shows that bottlenecks are defined as the road links where the arrival rate is near the maximum arrival rate expectation. In other words, the non-bottleneck roads can hold more traffic flow. Furthermore, they are supposed to have enough space to sustain the potential traffic increase caused by allocating police to bottlenecks. Therefore, the potential traffic increment *PTI* depends largely upon minimum traffic flow increase at the bottlenecks after deploying police. *PTI* of a bottleneck set *BS* could be calculated as Eq (18):

$$PTI=\underset{l\in BS}{\text{min}}\{u_{l}-[(1-p_{l})u_{l}+p_{l}w_{l}]\}$$ (18)

As the Eq (18) shows, the potential traffic increase of a path is the minimum potential increase of the bottleneck road link.

The potential flow increase of an evacuation path can affect the police resource utilization efficiency, while the traffic flow on the evacuation paths longer than that path also influence the police resource utilization efficiency index because we need to adjust the vehicles to the shorter paths. For each evacuation source, we choose the longest traffic-existing evacuation path as a baseline. The amount of traffic flow adjustment *TFA* is the traffic flow amount on the baseline path. In other words, we only adjust the flow of the longest evacuation path from a source to other paths with the same source.

The potential evaluation of an evacuation path should include both *PTI* and *TFA*. The following equation shows how to calculate the potential traffic flow improvement *PTFI* for an evacuation path.

PTFI=min{PTI,TFA} (19)

We estimate the potential traffic increase of a path to be the minimum value between the *PTI* and *TFA*. The time needed to calculate *PTFI* for each path is short, and *PTFI* can inform us in evaluating the evacuation paths.

The third procedure is allocating police. The police resource utilization efficiency index, which helps evaluate the evacuation paths heuristically, is defined in mathematical formulation. Allocating police should maximize the police resource utilization efficiency. We only allocate police to the most valuable bottleneck set. We determine which path will be allocated with the optimal solution of the program below:

$$\underset{path\in EP}{\text{max}}\;PRUEI=\frac{PTFI_{path}}{PC_{path}}$$ (20)

$$\text{S.T.}PC_{path}=\sum_{l\in BS_{path}}c_{l}$$ (21)

Additionally, *PRUEI* is the police resource utilization efficiency index; *EP* stands for the set of traffic-existing evacuation paths; *path* is an element of the set *EP*; *PC*_*path*_ is the police resource cost of allocating the police to the bottleneck set for that path. The *PRUEI* represents the return per police resource unit at this allocation, which is a heuristic indicator for evaluating the bottlenecked road. The solution of this program is the most valuable path, and we allocate police to the bottleneck set for that best path.

The final procedure is re-planning evacuation activity. In allocating police to valuable bottlenecks, the traffic capacity of the whole traffic network changes according to assumption 2). We need re-plan the evacuation activity to get an optimal evacuation plan under the new police allocation. We adjust some constraints of original ERPRA formulation as follows:

$$\text{min}Z=\sum_{i}e_{i}\sum_{j}\sum_{k}x_{ijk}t_{ijk}$$

S.T. (2)-(4), (6), (8)

$$\lambda_{l}^{L\text{max}}=u_{l},\;\forall l\in PRL$$ (22)

$$\lambda_{l}^{L\text{max}}=(1-p_{l})u_{l}+p_{l}w_{l},\;\forall l\notin PRL$$ (23)

*PRL* is the set of road links that have been allocated police officers. The program above is linear continuous programming. The Simplex Method could quickly find the optimal solution of the program. Procedures from a) to d) compose step 2, which updates the evacuation route and police allocation.

We loop step 2 until there are not any police resources remaining or until there is no additional decrease in total evacuation time. Clearly, the loop will stop when police resources are completely allocated. A non-decrease in evacuation time between the loop and the prior loop shows that the traffic capacity of whole network is almost saturated. It indicates that allocating more police resources is futile. Therefore, we can stop the loop and finish step 2 in both situations. The evacuation planning is performed until step 2 is finished.

## Computational Results

### Experimental design

**Fig 2:**
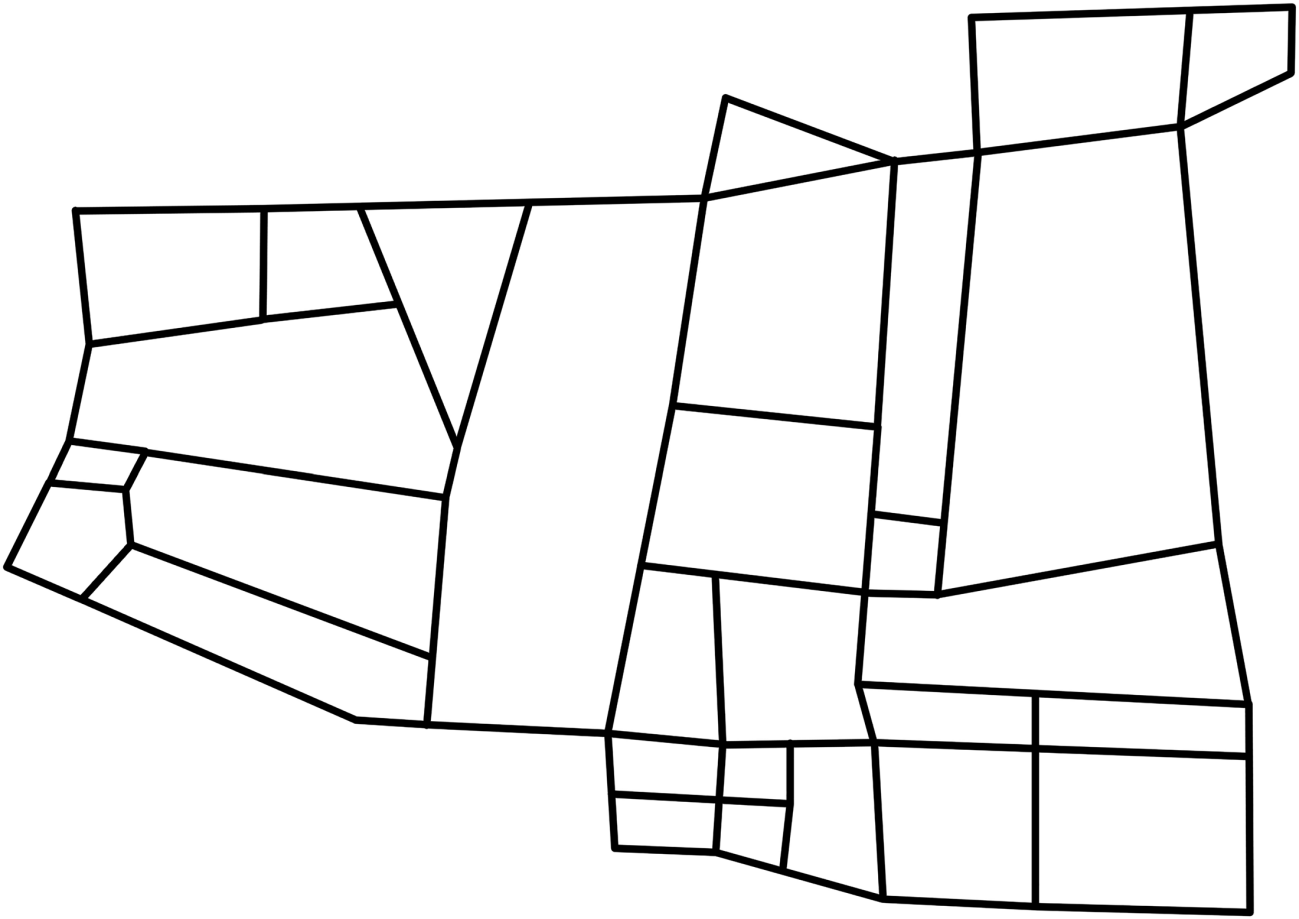
Transportation road network of Changsha, China.

**Fig 3:**
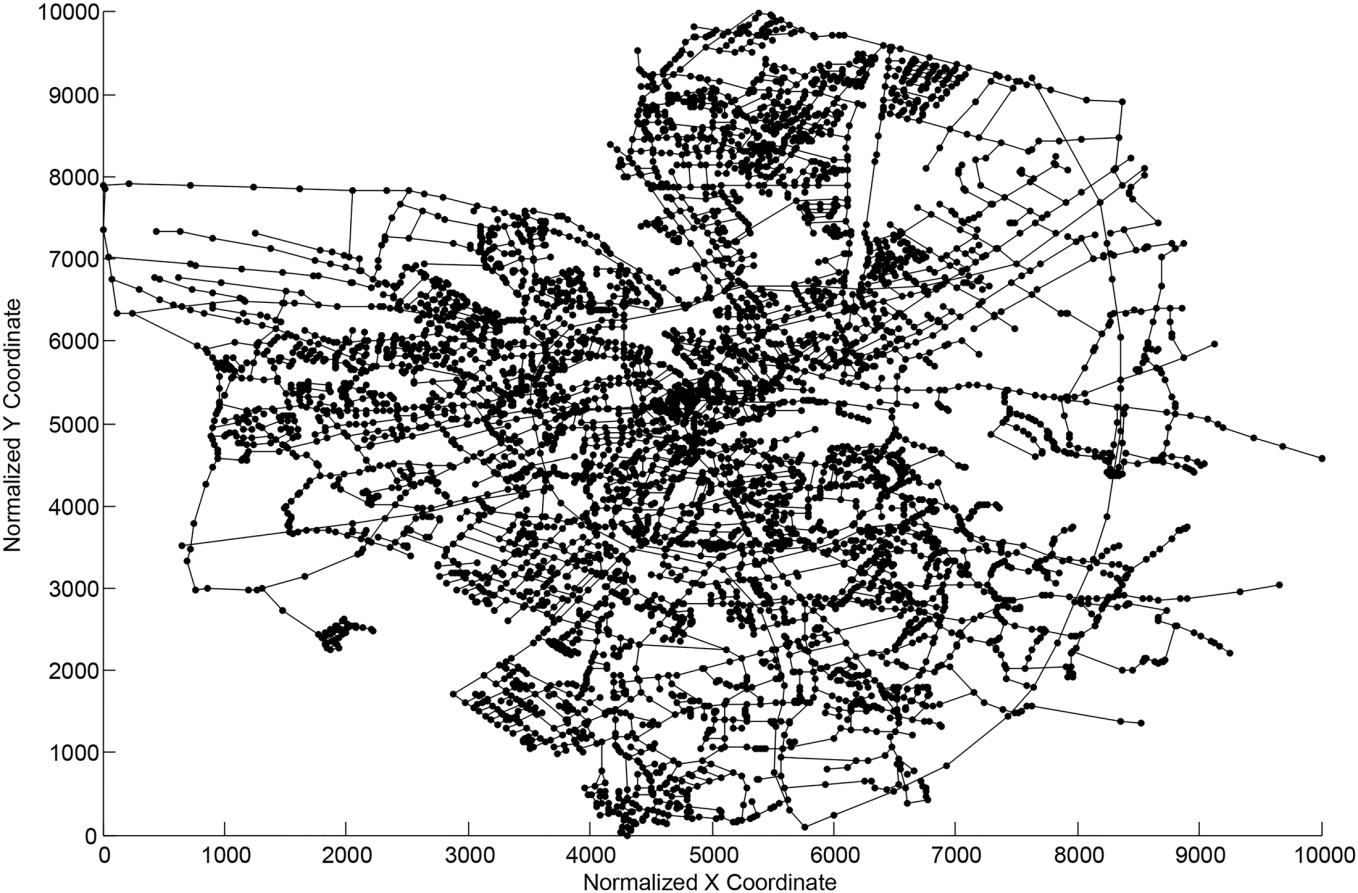
Transportation road network of Oldenburg, Germany.

In this section, we tested the performance of the two ERPRA solution algorithms on different datasets. Some of the datasets are based on the real transportation network of Changsha, China, as shown in Fig 2 (the original GIS data is from openstreetmap.org [43]). Other datasets are based on the subnet of the road network of Oldenburg, Germany, as shown in Fig 3 [44]. Specifically, (1) geographical information, such as the number (*N*) and length of the roads, is calculated by a commercial GIS software, ArcGIS, based on the data of the two cities; (2) *k*-th shortest paths parameters, such as *t*_*ijk*_ and *α*_*lijk*_, are derived during preprocess using mature methods [9–11] based on geographical information; (3) other related parameters, including *e*_*i*_, *v*_*j*_, *λ*_*i*_, $\lambda_{l}^{L\text{max}}$, *u*_*l*_, *w*_*l*_, *p*_*l*_, *r*_*l*_, *c*_*l*_, and *B*, are randomly generated (See S1 Dataset). Randomly generated transportation networks (See S2 Dataset) were also used to test the performance of the two algorithms. The unit of objective value is a unit time, but comparing the objective value across network scales is meaningless due to random parameters of different networks. The algorithms were coded in MATLAB by invoking CPLEX Optimizer v12.6. We executed the programs on a 32-bit Windows 7 computer with a 2.1GHz Duo core CPU and 1.0 GB of physical RAM.

### Comparison of the two solution methods

To compare the performance of the heuristic algorithm and the linearization method, a series of experiments were conducted on the same datasets and with the same computer.

#### Different scales of the real cases

In order to verify the feasibility of the two methods, we tested the algorithms on differently sized real road networks (see S1 Dataset). The number of evacuation sources of the networks is 4, and the number of destinations is 3. Table 1 shows the performance of the heuristic method and the linearization method. The gap represents the objective value difference between the two algorithms and then divide the objective value of heuristic.

**Table 1:** The two algorithms comparison on different network scales.

		Objective Value			Solving Time (s)	
*N*	*L*	Heuristic Method (unit time)	Linearization Method (unit time)	Gap ^a^	Heuristic Method	Linearization Method (s)
46^b^	154	1859541	1854465	0.0027	2.806	41.871
204	448	186076	184670	0.0076	14.529	387.226
395	806	324137	-^c^	-	7.064	-
735	1626	514295	-	-	24.694	-
1448	3473	329669	-	-	18.165	-
1852	4400	252504	-	-	15.718	-
3118	7276	386026	-	-	22.719	-

^a^ Gap = (objective value of heuristic-objective value of linearization)/objective value of heuristic

^b^ The first dataset is of Changsha, China, and others are of Oldenburg, Germany

^c^ “-”stands for out of memory.

According to Table 1, both of the methods can handle the evacuation routing and police deployment problem. According to networks of 46 and 204 nodes, the linearization method and the heuristic algorithm are able to figure out results. Furthermore, the gaps between the two methods are below 0.8%, which can be ignored. This suggests that the optimality of two algorithms is almost the same. It also cross-validates the correctness of the two methods. The heuristic’s calculation time is much less than that of the linearization, with the latter taking over 20 times longer than the former. It is clear that the heuristic algorithm is significantly faster than the linearization algorithm. Table 1 also indicates that linearization method fails to handle moderately size cases due to insufficient memory. A sharp increase in solving time from 41.871 s to 387.226s is seen when number of nodes grows from 46 to 204. On the contrary, the expanded scale does not lead to increases in heuristic calculating time. It is noticed that the heuristic method could solve the problem with 3118 nodes and 7276 road links, which shows the real-world applicability of the heuristic method. This implies that different mechanisms lead to different performance benefits.

#### Different scales of random cases

**Fig 4:**
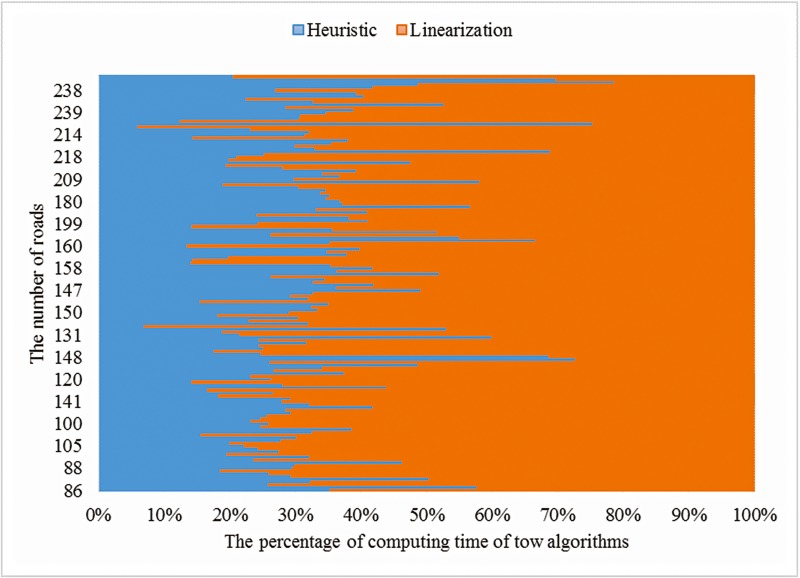
The computing time of heuristic algorithm and the linearization method.

**Fig 5:**
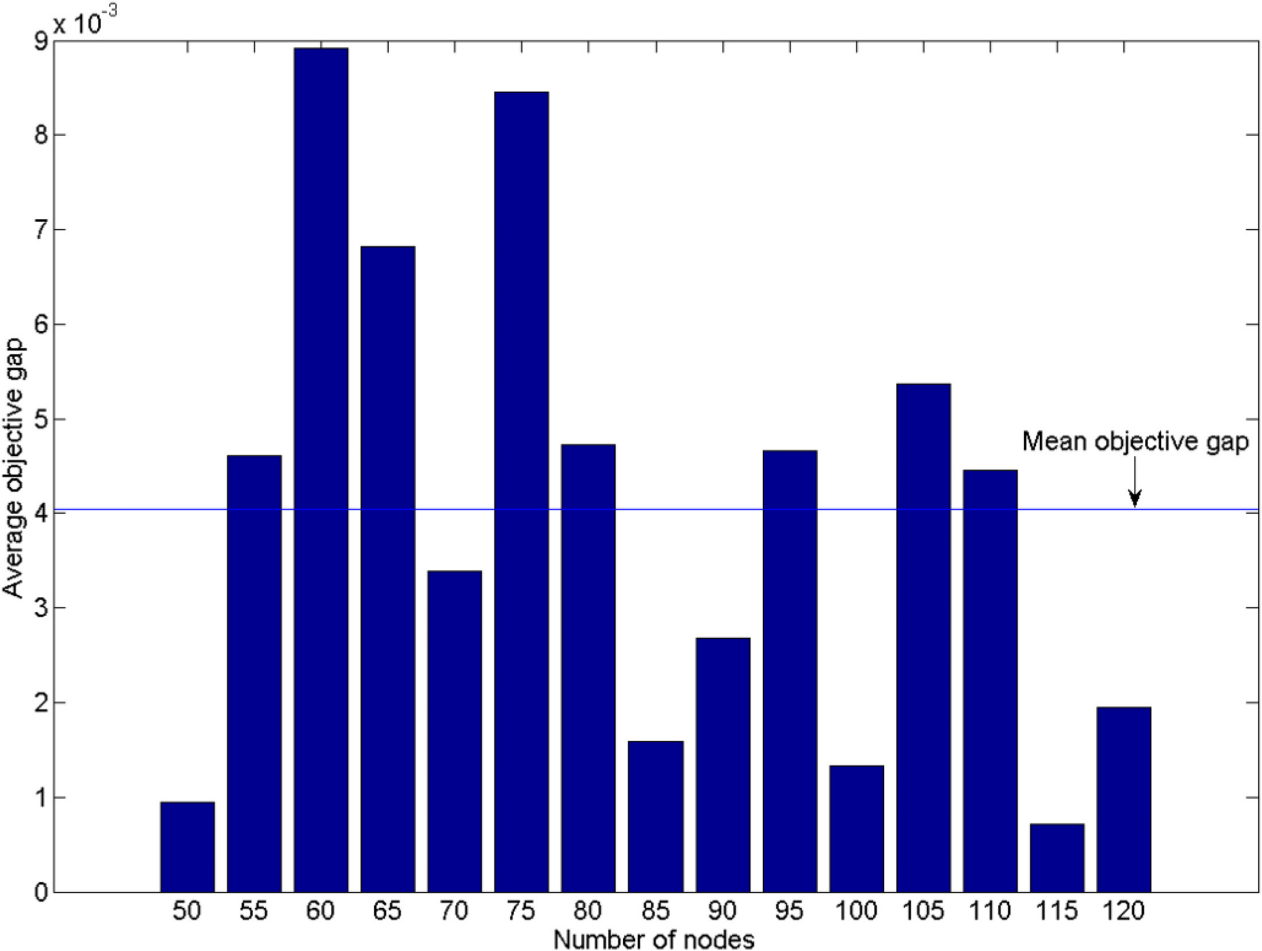
The objective gap difference of the two methods.

Random experiments provide more insights into the effect of network size on the two methods over a large number of experiments. We generated random transportation networks and randomly set up the parameters (see S2 Dataset). Then, two methods were tested based on the random data. Results of the random experiments are shown in Figs 4 and 5.

Fig 4 shows the calculation time for the two methods in each random case. The cases feature different numbers of nodes ranging from 50 to 120, and the number of road links ranges from 86 to 238. And the number of roads is positive correlation to the number of nodes, so we take the former one to represent the scale of the networks. In order to compare the computing time of two algorithms in different network scales, we normalize the sum of computing time of two algorithms for each network scale. When the scale of networks extending more than 120 nodes and 238 roads, the linearization method solved with CPLEX is interrupted by a memory error, but the heuristic method continues to work. On average, the calculation time of the linearization method is 2.619 times greater than that of the heuristic. The heuristic method performs more efficiently than the linearization method for 88.67% of all network scales.

There are two reasons leading to the solving time difference between the heuristic method and linearization method. First of all, linearization method needs to solve a scale-expansion optimal problem. As mentioned in section 4.2, the linearized procedure adds numerous extra variables and constraints in order to match the original program, and the exact number of extra variables and constraints is linked to number of nodes and road links. Therefore, increasing the size of the road network causes considerable linearization model scale growth, which increases the calculating time of the linearization method. On the other hand, the number of calling linear continuous program solver of the heuristic method is much less than the linearization’s. The LERPRA model is a linearized mixed integer program, which is solved by CPLEX. And CPLEX solver makes use of the algorithms similar to branch-and-bound method to solve this kind of problem. It is known that branch-and-bound method needs call linear continuous program solver over and over again, until the solution of linear continuous sub-problem satisfied the binary constraints of primary problem (here is LERPRA problem). In the worst case, the times of solving sub-problem could be exponential, namely, 2^*L*^ in the LERPRA problem. Whereas, the time consumption of the heuristic largely depends on the number of loops performed. And the main time consuming of each loop is solving a linear continuous problem. The number of loop executions hinges on police budget and the success of iterative improvement. That is to say, the number of calling linear continuous solver at most is *B*. Simply, if we tentatively treat the computing time of linear continuous solving procedure as a basic time consuming unit (in reality, the sub-problem of LERPRA problem is more time-consuming than the optimal problem in each iteration of the heuristic method), then, the time complexity of the linearization method would be *O*(2^*L*^), however, the heuristic would be *O(B)*. Consequently, the heuristic method performs well in terms of time consuming, when expansion of the network scale is the isolated variable.

Fig 5 depicts the objective gap of the two methods. The results are divided into several groups according to the number of nodes in the transportation network. The average objective gap, overall, is nearly 0.004, and the maximum gap is 0.009. This indicates that the effectiveness of the two method is almost the same. Furthermore, the gaps fluctuate with network scale. There is no distinct relationship between gap and scale (i.e., when network grows, the optimality of heuristic method is excellent). On one hand, the heuristic method is suitable in large networks because it saves much time and retains good optimality. On the other hand, if the size of network is small enough and the calculation time of linearization method is acceptable, the linearization method would be the best choice because the result of linearization is always optimal.

#### Different numbers of evacuation sources and destinations

Apart from the scale of the network, the number of evacuation sources and destinations might have an impact on the solving time. We conducted the experiment on the same road network N = 46, L = 154 (See S1 Dataset), except that the number of evacuation sources and destinations varied. The results are shown in Table 2.

**Table 2:** The two algorithms comparison on different number of sources and destinations.

		Objective Value			Solving Time (s)	
Num. Sources	Num. Destinations	Heuristic Method (unit time)	Linearization Method (unit time)	Gap	Heuristic Method	Linearization Method
1	1	435332	435331	0.0000	0.313	0.812
2	2	971449	971449	0.0000	1.177	2.013
2	3	875706	875706	0.0000	1.391	3.105
4	3	1859541	1854465	0.0027	2.806	41.871

Table 2 shows that the solving time of both algorithms have a positive correlation with the number of sources and destinations. The solving time for the heuristic increases more slowly than the solving time for the linearization method does. The solving time of the linearization method jumps sharply when the number of sources and destinations is 4 and 3, respectively. In contrast, the heuristic method’s solving time grows slowly. This phenomenon reflects the differences in sensitivity to number of sources and destinations between the linearization method and the heuristic. With the exception of adding more parameters and variables, increasing the number of sources and destinations leads to an increase in only the constraints (2) and (3) in original formulation. The linearization formulation must add many constraints to ensure equivalency to the original model. Adding one source would lead to add L*J*K variables *z*_*lijk*_ and add 4*L*J*K constraints about *z*_*lijk*_. From the analysis in Section 5.2.2, extending the scale of LERPRA program would increase the solving time of sub-problem so that it gives rise to a considerable increase in solving time of the linearization method. By contrast, the heuristic method does not extend much scale of the original program. Adding one source only cause adding J*K variables *x*_*ijk*_ and adding 1+J*K constraints about *x*_*ijk*_. As a result, it shows great tolerance to increasing numbers of sources and destinations. Theoretically, when the number of sources and the number of destinations are close, the effect of adding a source or a destinations are almost the same. In addition, the objective gap between the two could be ignored, which shows the reliability of the heuristic method in terms of optimality.

#### Different traffic accident probabilities

The traffic accident probabilities might distribute the evacuation vehicles because the higher traffic accident probabilities are, the less vehicles the roads can hold. Therefore, the evacuation plan might be affected by the traffic accident probabilities. The dataset of Changsha was used for testing the influence of accident probabilities (see S1 Dataset). We changed the maximum traffic accident probabilities *r*_*l*_ of each road link by multiplying a coefficient. The objective gap and the solving time of two algorithms were both recorded.

**Fig 6:**
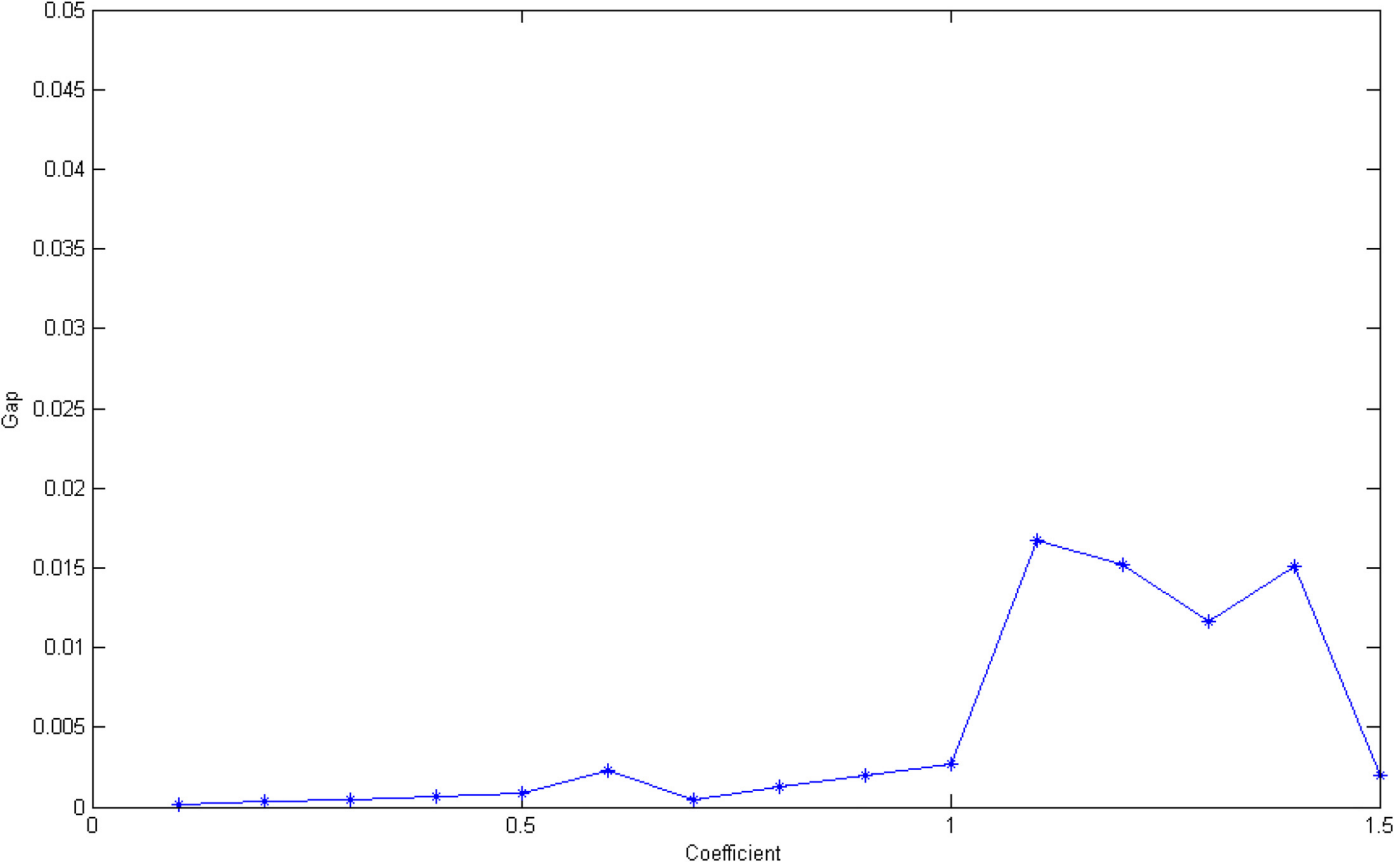
The line graph of accidents probabilities coefficients and gap.

Fig 6 presents the trends in gap between the two methods while traffic accident probabilities increase. When the coefficient is less than 1, the gaps are all below 0.3%. After peaking at 1.1 (where the gap is 1.7%), the gap tapers off to approximately 1%. Eventually, the gap falls back to approximately 0.25%. Although that gap is still acceptable, the fluctuation is relatively large. An increasing coefficient indicates that the capacity of the roads is decreasing and that the number of bottlenecks might soar. Considering the sequential nature of the police allocation used by the heuristic method, a bad choice of deployment in an iteration might lead to a slip into a local optimum solution. The heuristic method might be not useful for solving a problem with numerous bottlenecks.

**Fig 7:**
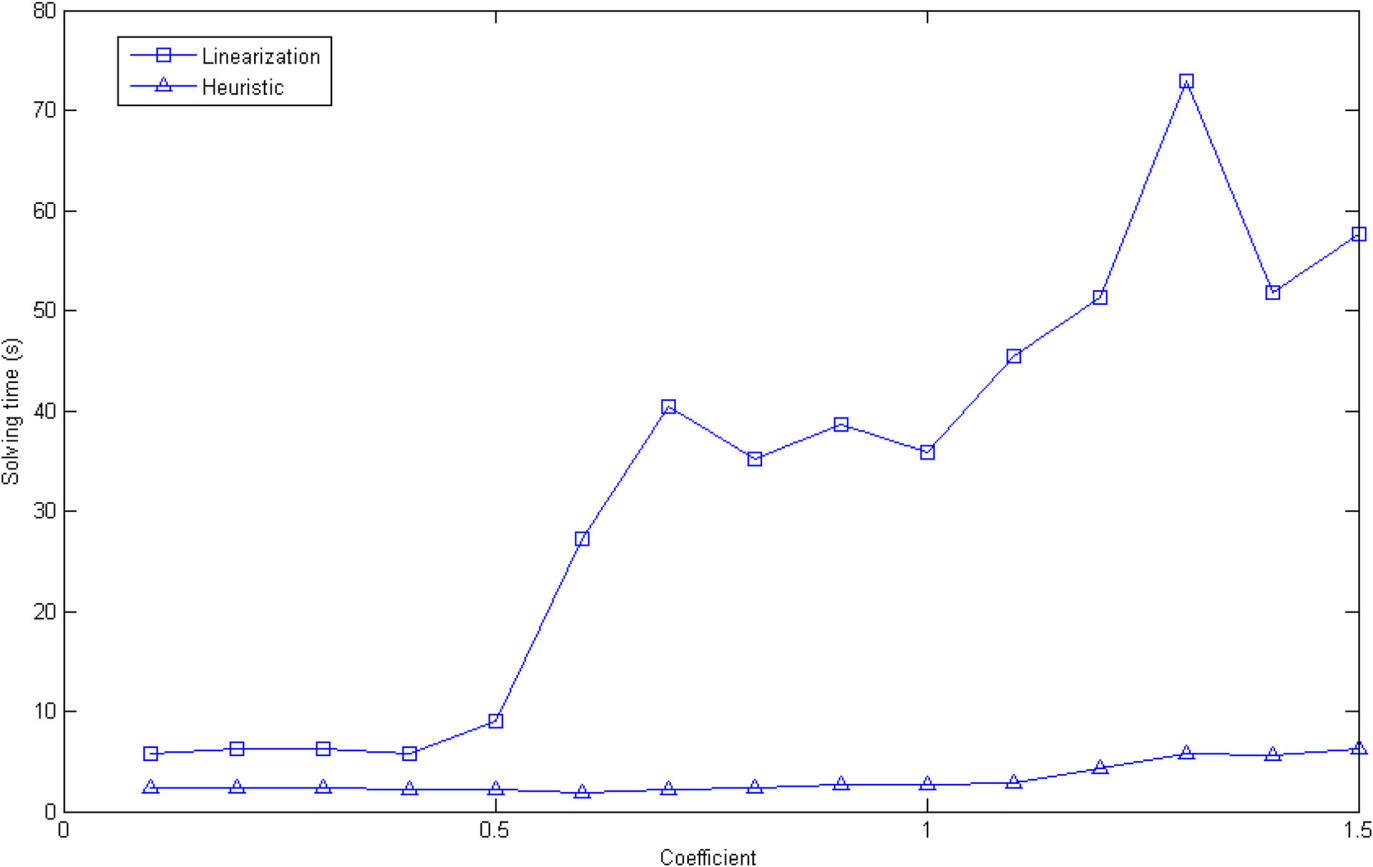
The comparison solving time (s) of two algorithms with coefficient varying.

Fig 7 suggests that the solving time of the two methods increases while the accident probabilities grow. However, the linearization method’s solving time changes more drastically than the heuristic’s does. For instance, with a coefficient of 1.5, the linearization method takes nearly 60 s, while the heuristic takes only 7 s. The reason why the linearization method is sensitive to accident probability might involve the recognition that the spreading traffic flow creates more *x*_*ijk*_ that are beyond 0. Consequently, many constraints, such as constraints (12), could not be cut off by CPLEX solver. In a sense, the real program scale, which was preprocessed by solver, grows considerably. So the higher the accident probability is, the longer the linearization’s solving time is. Without extra constraints and variables, the heuristic method performs relatively steadily in terms of solving time.

### Rate of return on police resources

Emergency managers care about assessing the rate of return on police resources. Police resource return analysis not only shows the effectiveness of police resources but also provides a reference for police budget preparation. This analysis takes the road network of Changsha as an example (See S1 Dataset). Parameter *B* ranges from 0 to 35, and we record the resulting objective values.

**Fig 8:**
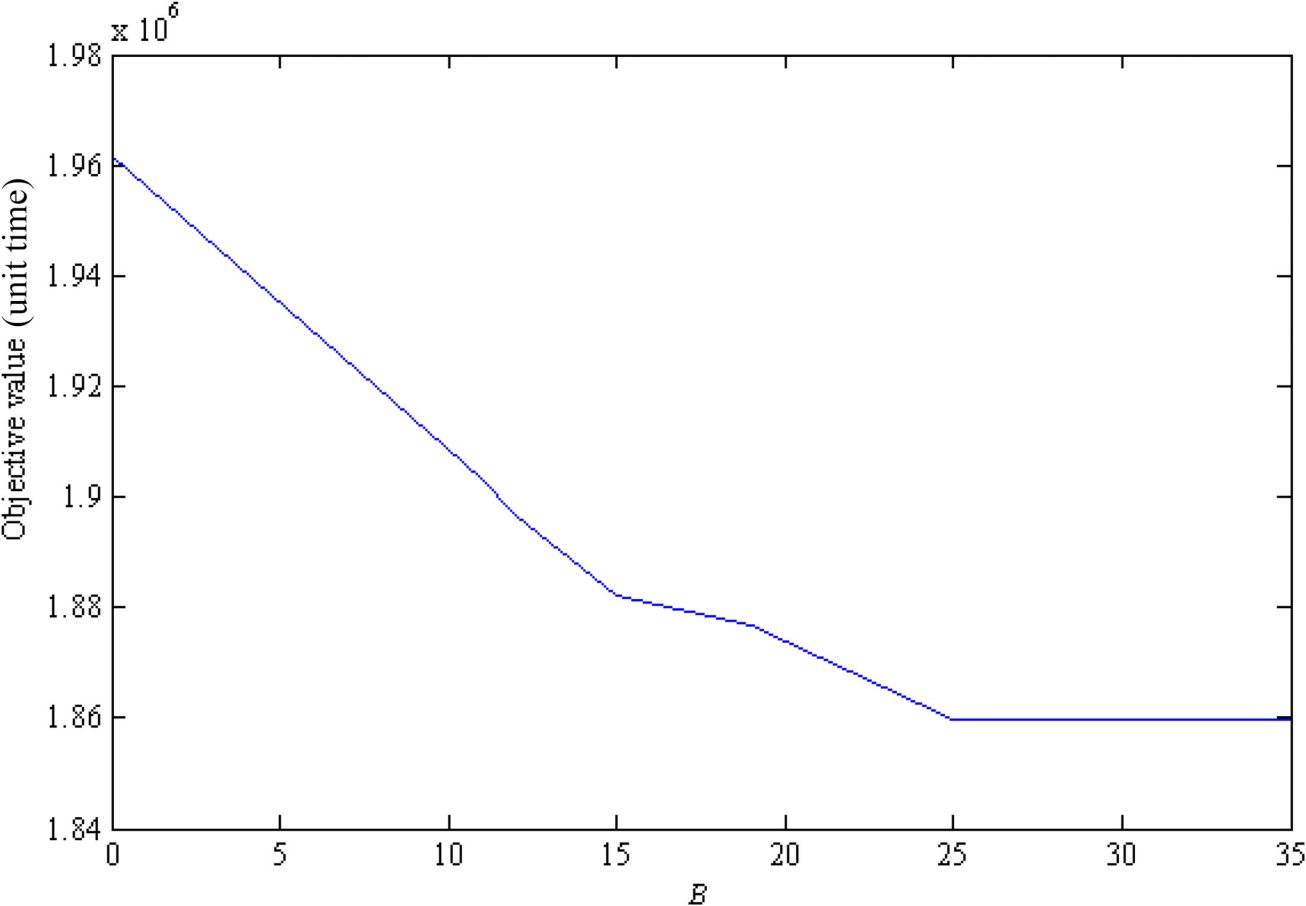
The relationship between objective value and police resource budget.

Fig 8 depicts the objective value while police budgets grow. Without police, the overall evacuation time is considerably high, over 1.96 million units. Provided that the police budget is sufficient, the total evacuation time decreases by 100,000 units. Because every second in evacuation is precious, this decrease speaks volumes to the effect of police on evacuation. The total evacuation time declines quickly during a police resource shortage stage. When police resources are relatively adequate, more police resources seem to make no sense. We see that the derivative of the curve decreases progressively. That is, the rate of return on police resources is diminishing; there is only a marginal reduction as the police budget continue to increases.

Additionally, having sufficient police resources reduces the evacuation clearance time by approximately 5.21% compared with zero use of police resources. It should be noted that the 5.21% decrease is comparing with the clearance optimal evacuation flow without police. In evacuation, the load of transportation network is extremely heavy, increasing traffic flow even a little is remarkable. And, the objective value represents the total evacuation time spent on roads, which excludes the waiting time before evacuees enter into the road networks. Therefore, the 5.21% decrease in evacuation time implies a reduction in waiting time, which reassures flustered evacuees. Furthermore, it reduces the congestion of roads and the difficulty of emergency management. To sum up, the 5.21% decrease of evacuation clearance time makes big difference.

## Conclusions

In this paper, we present a NMIP model to describe the integrated police resources allocation and emergency evacuation routing problem. The integration optimization model allows decision makers to deal with complex combinatorial optimization problems, including police resources allocation and evacuation routing. Two algorithms are presented. One is a linearized model, which uses extra variables and constraints to maintain equivalency to original model, and it can be solved with CPLEX solver. The other is a heuristic method based on the police resource utilization efficiency index, and it is designed to generate a near-optimal solution to the problem. Numerous computational examples with different datasets are conducted to verify the capabilities of the two methods, and these examples indicate that the heuristic method is more efficient than the linearization method in the majority cases. And the heuristic method could solve the problem with larger scale than the linearization could. However, the linearization method shows better optimality than the heuristic in some cases. Sensitivity analysis of police resources indicates that too many police resources for a given evacuation network is unnecessary. In the future, in order to improve the flexibility and robustness of the evacuation planning, we will extend our work to a dynamic model. In addition, we will change the linear relation between vehicle numbers and accident probability into a non-linear relation to make the model more accordant with the practical traffic situation.

## Supporting Information

S1 Dataset The real network dataset. (ZIP)

S2 Dataset The random network dataset. (ZIP)
